# Supplementary figures and images for: Isolation and screening of fungi for enhanced agarwood formation in Aquilaria sinensis trees
Source: PLoS One. 2024 Jun 14;19(6):e0304946. doi: 10.1371/journal.pone.0304946 (PMC11178209; doi:10.1371/journal.pone.0304946)

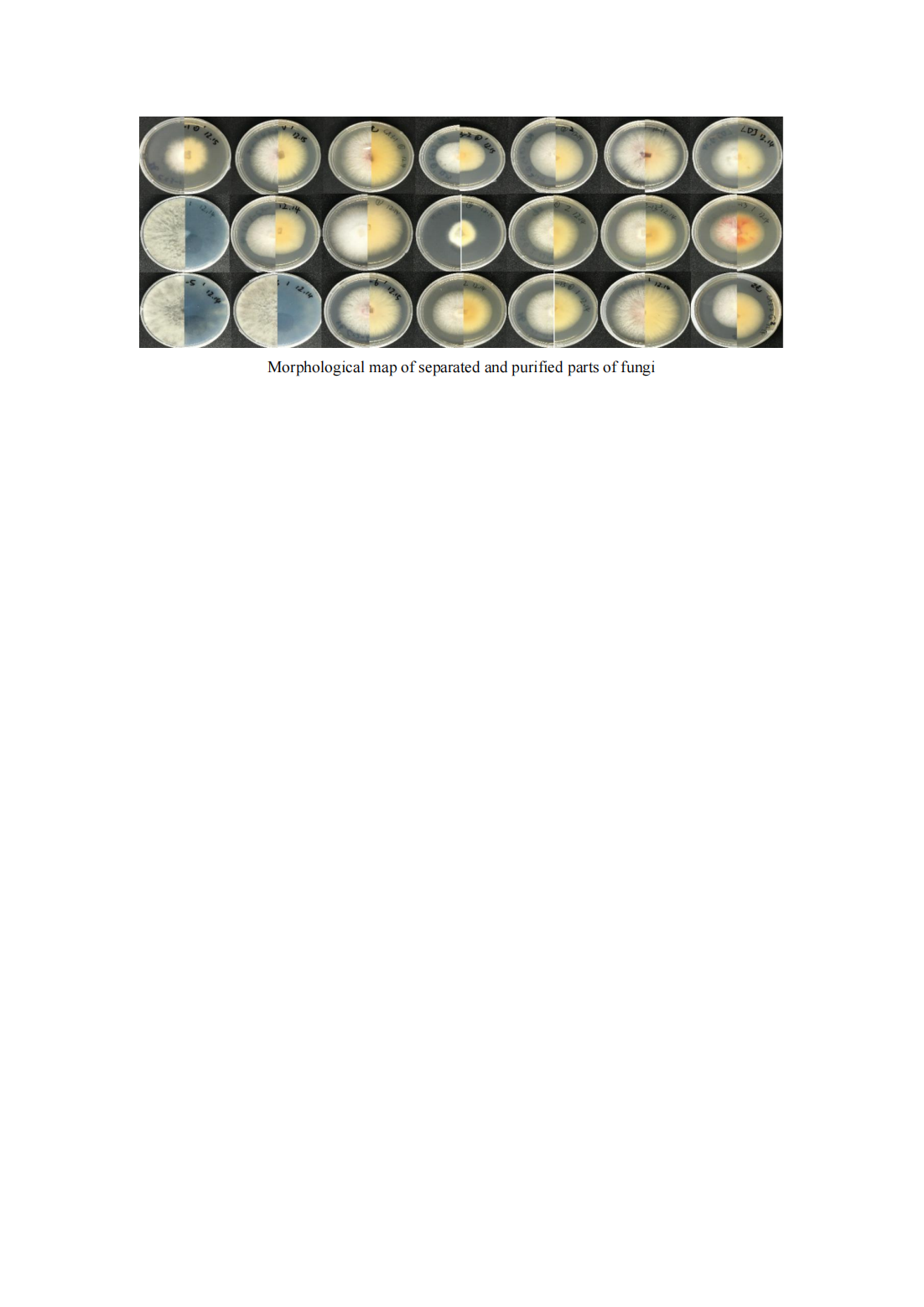

Supplement: S1 Fig — (TIF) [file pone.0304946.s001.tif]

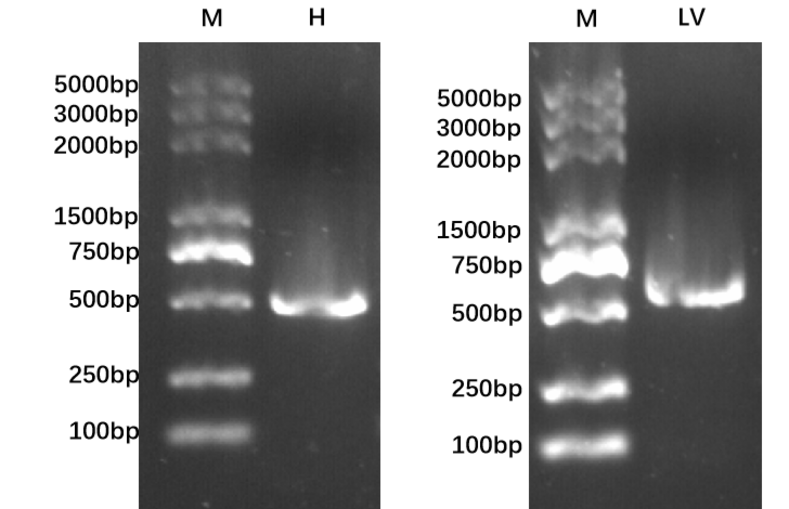

Supplement: S2 Fig — (TIF) [file pone.0304946.s002.tif]

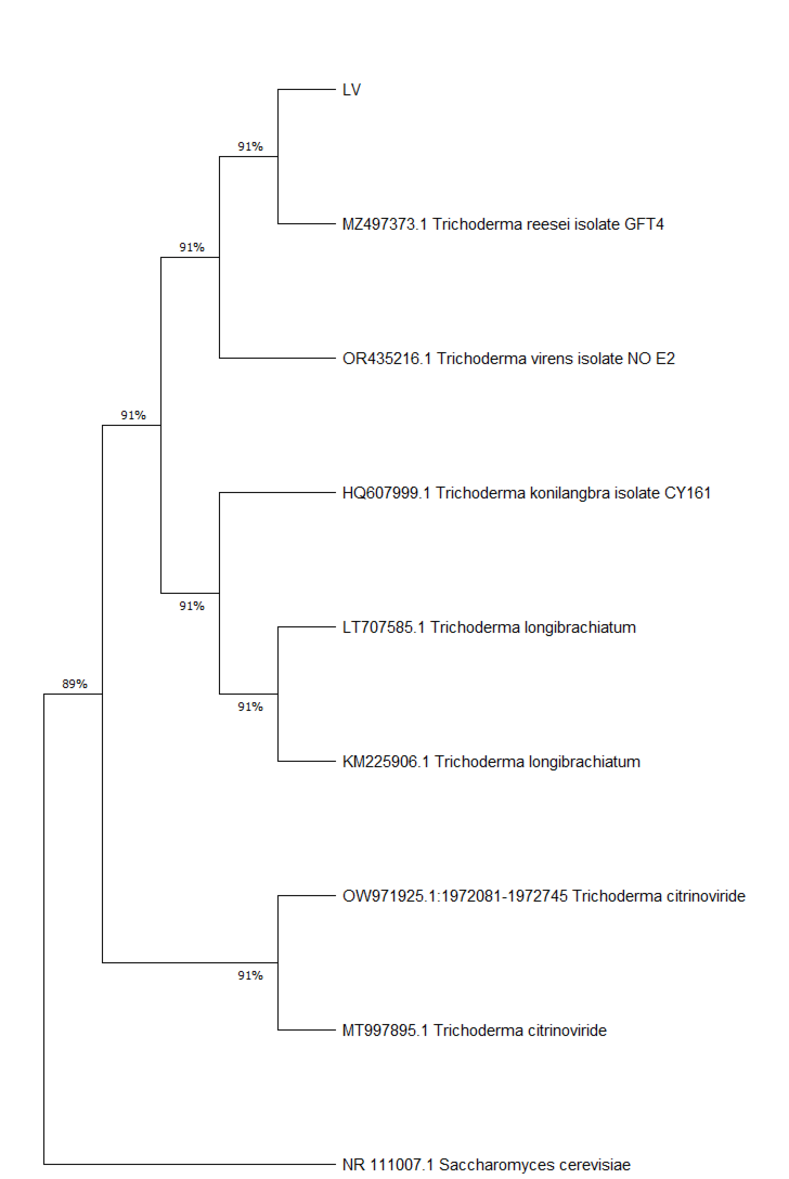

Supplement: S3 Fig — (TIF) [file pone.0304946.s003.tif]

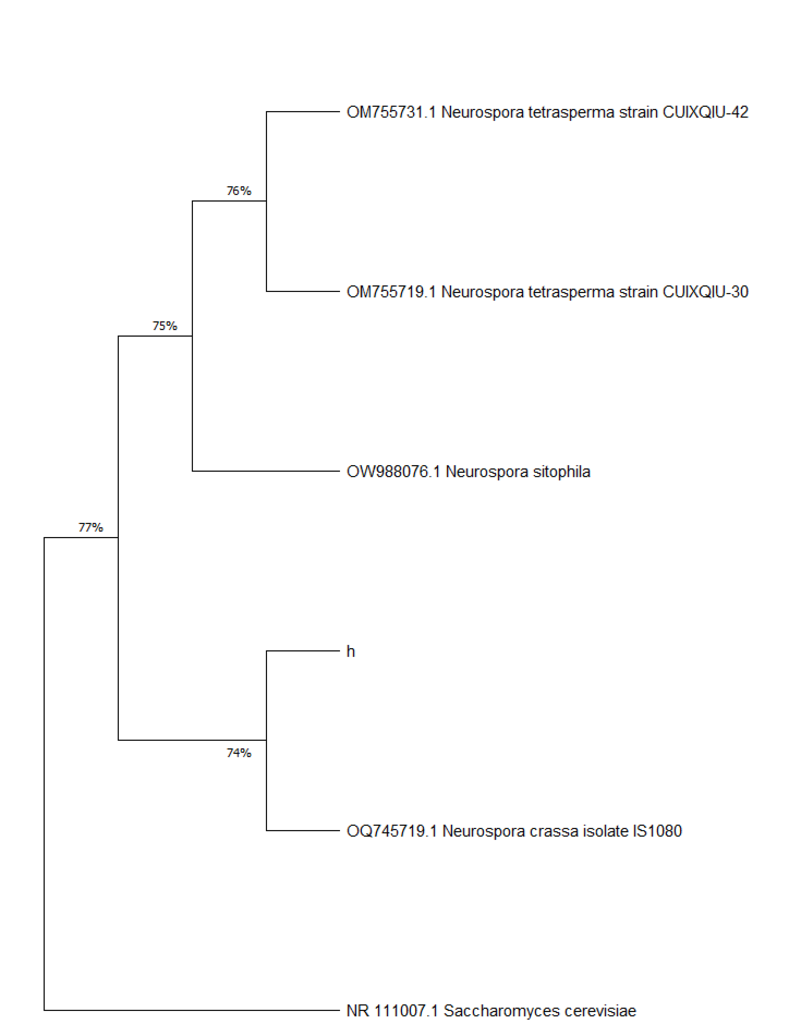

Supplement: S4 Fig — (TIF) [file pone.0304946.s004.tif]

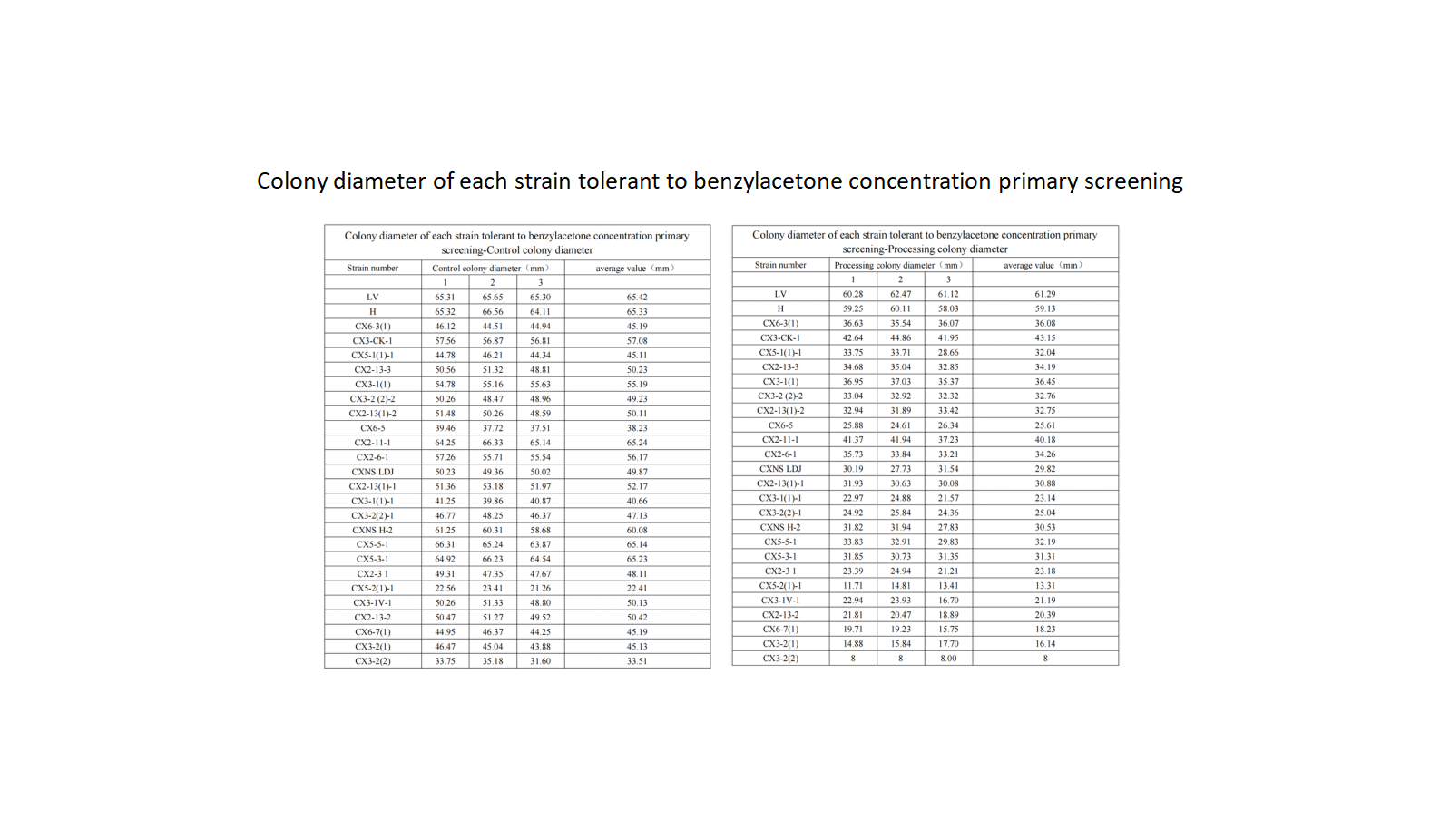

Supplement: S1 File — (TIF) [file pone.0304946.s005.tif]

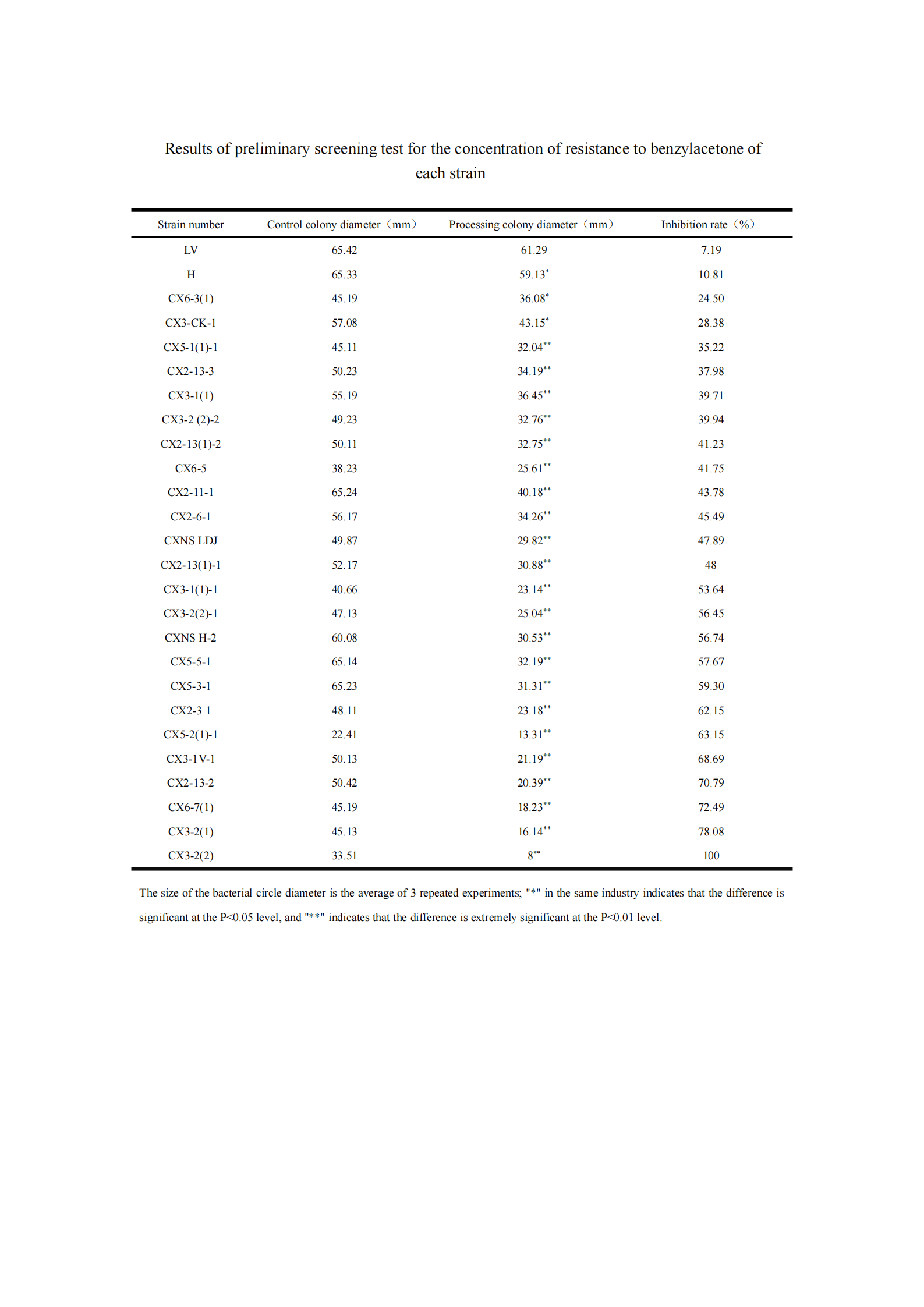

Supplement: S1 Table — The size of the bacterial circle diameter is the average of 3 repeated experiments; "*" in the same industry indicates that the difference is significant at the P<0.05 level, and "**" indicates that the difference is extremely significant at the P<0.01 level. (TIF) [file pone.0304946.s006.tif]

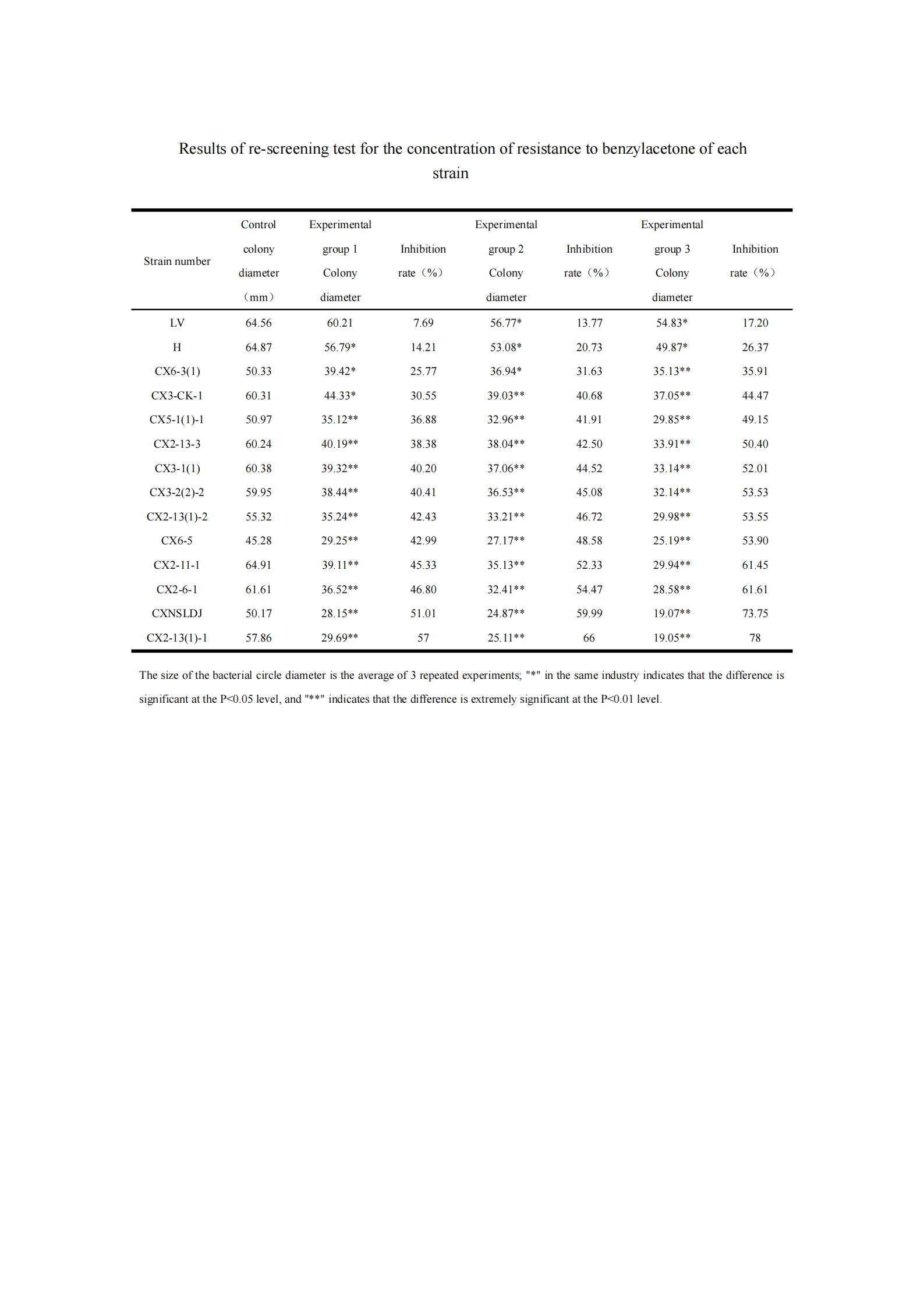

Supplement: S2 Table — The size of the bacterial circle diameter is the average of 3 repeated experiments; "*" in the same industry indicates that the difference is significant at the P<0.05 level, and "**" indicates that the difference is extremely significant at the P<0.01 level. (TIF) [file pone.0304946.s007.tif]

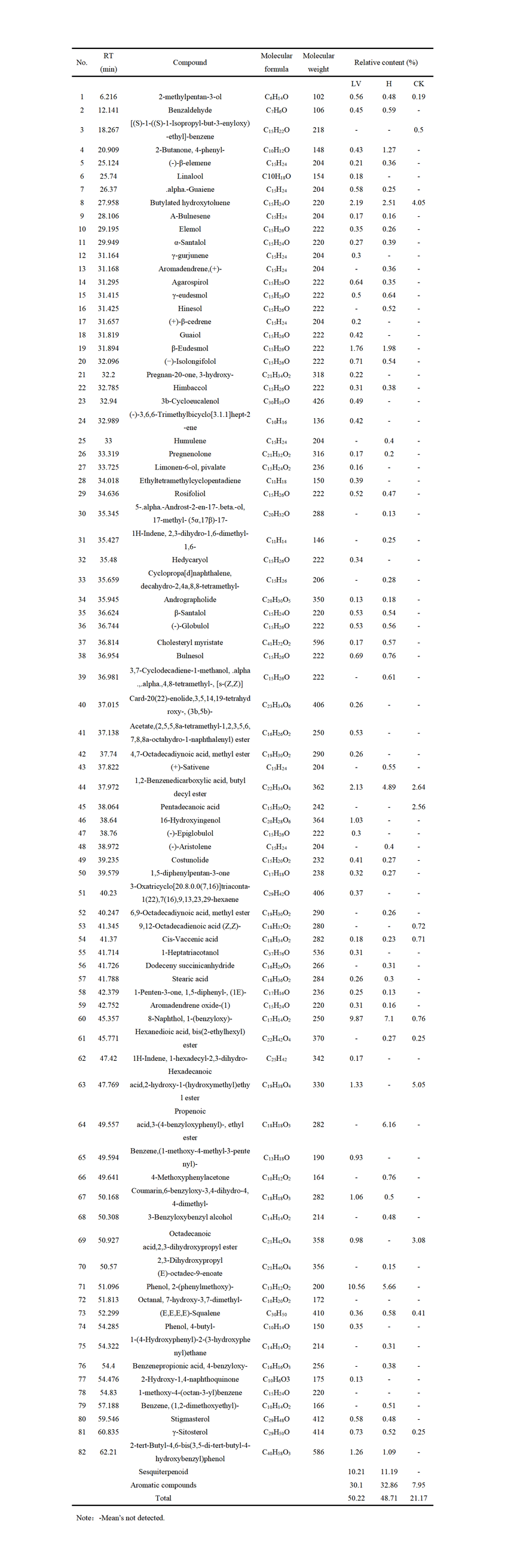

Supplement: S3 Table — Mean’s not detected. (TIF) [file pone.0304946.s008.tif]
