# Supplementary material for: Isolation and screening of fungi for enhanced agarwood formation in Aquilaria sinensis trees
Source: PLoS One. 2024 Jun 14;19(6):e0304946. doi: 10.1371/journal.pone.0304946 (PMC11178209; doi:10.1371/journal.pone.0304946)
Supplement: Attachment — Submitted filename: Response to Reviewers.docx [file pone.0304946.s009.docx]

Response to Reviewers

According to the review comments from 2 editors, this paper has an overall problem with the English language, so I made a complete revision of my manuscript to meet the journal requirements.

Reviewer #1

1、Why were stains screened for resistance using benzylacetone?

Studies on endophytic fungi growing on medicinal plants have shown that endophytic fungi can not only synthesize their own active components, but also have the ability to promote the synthesis of active components by the host plant . Fungi promoting the formation of agarwood are firstly tolerant to some extent to the active ingredients of agarwood. The composition of agarwood is complex, consisting of three main components: sesquiterpenes, 2-phenylethylchromones, and aromatic compounds. Benzyl acetone is a representative compound of the aromatic content in agarwood .

2、“The Trichoderma sp. and Neurospora sp. samples yielded ethyl ether extracts of 3.62% and 4.04%, respectively” what significance does this statement has?

“The Trichoderma sp. and Neurospora sp. samples yielded ethyl ether extracts of 3.62% and 4.04%, respectively” In Chinese literature, most of them will write this "extraction rate" to express the accuracy of the extraction method. This has been modified in the text.

3、The sum of the relative contents of the aromatic components was 30.1% and 32.86%, respectively. What significance does this statement has? Statement that make characteristic contribution to the results need to be highlighted in abstract.

These two agarwood-specific chemical constituents accounted for a large proportion of the total chemical composition, which showed that the generated agarwood was of good quality.

4、The relative contents of sesquiterpenes were 10.21% and 11.19%. Trichoderma sp., Neurospora sp. were able to effectively induce the production of agarwood within 6 months, and their good effect on agarwood formation and shortening the time of agarwood formation. Not clear what new information/ knowledge or method has been created by this study.

In this study, pretreatment was carried out before exposing the Aquilaria sinensis trees to fungi. Moreover, isolated fungi were screened for resistance against benzyl acetone, which is a major component of agarwood. Artificial inoculation of fungi in Aquilaria sinensis trees was able to effectively induce the formation of agarwood over a 6-month period, The produced agarwood incense were analyzed by gas chromatography-mass spectroscopy (GC-MS). The method presented here is able to narrow down the range of fungi that promote agarwood formation, and reduce the time and monetary cost of screening these fungi. The study would expand the library of fungi known for promoting agarwood formation.

5、What does the statement “It provides effective support for the expansion of the fungal library that promotes agarwood formation” substantialize or conclude? Choice of word usages have diluted the real meaning of the statement.

Changes have been made in the text

6、What is Introduce? Should be Introduction.

Changes have been made in the text

7、Its trunk does not produce agarwood if there is no external damage, only after natural or man-made injuries such as slashes, injuries and diseases, insects and ants, lightning strikes, etc….is a incomplete sentence

Changes have been made in the text

8、Agarwood is mildly warm in nature. How??

Changes have been made in the text. In Chinese medicine, agarwood is mild in nature.

9、Since when Agarwood has been used as Precious spice? Please quote references.

Changes have been made and references have been cited

10、Historically, China's agarwood resources are very rich, with the discovery of the medicinal value of agarwood ……………………….. field of Aquilaria sinensis has been very little, was sporadic distribution. Is a very complex statement and doesn’t make a good read. This needs simplification and usage of proper English to convey the core thought.

Changes have been made in the text

11、11.There have been many publication on artificial inoculation in A sinensis recently, a comparative account of the same would have been more interesting read which could have highlighted upon the advantages of the method devised in the study.

Changes have been made in the text
12.What is stained wood?

Poorly worded, should be wood that changes colour
13.Why Benzylacetone was selected as a resistance indicator needs to be elaborated? What added advantage it confers to the study is not apparent.

Changes have been made in the text
14.Justification for carrying out the study in not adequate in view of the available already.

Changes have been made in the text
15.The method used is amalgamation of too many method Physical, heat, electric current creating a state of over stress to the plant , where within under humid condition any fungal pathogen attack on the bark is possible. The fungus growing may not necessarily be endofungal community specific to the plant. A statement or clarification in this regard would be welcome by readers.

At first, we were trying to take endophytic fungi after fire-burning Aquilaria sinensis trees, but it was interesting to observe that the epidermis of Aquilaria sinensis trees was covered with fungi, and then we wanted to start the experiment with that colony. It is not said that the fungi covering the epidermis of Aquilaria sinensis trees are endophytic fungi.
16.The prepared sample was weighed 3 g wet weight into a 50 mL centrifuge tube and 15 mL of ether was added to extract the sample ………………………………….to obtain the ether extract, the extraction was repeated three times, then the extracts were combined and weighed in a fume hood after drying and the volatile oil content was calculated. How was it calculated? Statement also needs restructuring to make it easy to understand and read.

Changes have been made in the text
17.Table 1 showing that benzylacetone produced varying degrees of inhibition on all 26 isolated fungal strains can be a shown as supplementary data.

Changes have been made in the text
18.What is LV, H and CK need to be introduced to readers more properly in a one statement.

Changes have been made in the text
19.The discoloured wood was subjected to chemical compositional identification and the yield of ethyl ether extract was 3.62%, 4.04% and 1.94% for LV, H and CK respectively. Its GC-MS total ion flow (Figure 5). What does this statement mean?

Changes have been made in the text
20.“signifying the relative stability of the sesquiterpenes” How? Elaborate.

This paper would like to express that in this study sesquiterpenoids are more stable as compared to aromatic compounds.
21.Which lacked any of the 3 major active chemical components of Agarwood. What are these major active chemicals name them here.

Changes have been made in the text
22.The comparative analysis of the data in form of a discussion on the new knowledge gained in background all the existing knowledge is totally lacking.

Changes have been made in the text
23.Discussion of results is missing. That makes the manuscript sound vague. There is no reference to previous work in same field. There is basic data but very poorly presented. No scientific reasoning for the experimentation could be found in the text.

Changes have been made in the text
24.Table 1 and 2 should be made supplementary data.

Changes have been made in the text
25.Figure 3 should be made supplementary data.

Changes have been made in the text
26.Figure legends for Figure 4 and 5 poorly presented. Figure legend for 4 is missing.

Changes have been made in the text
27.The conclusion is confusing. What is the conclusion of the study?

Changes have been made in the text
28.“The fungi selected in this paper have good effects in promoting Agarwood production and shortening Agarwood formation time in Aquilaria sinensis trees” How? Prove the statement by comparing with previous available data needed in text.

Changes have been made in the text
29. Fusarium and Trichoderma are popular choices for inoculation, the study should list out the novelty of the present work more clearly.

Neurospora sp. sap was used first time in China to induce the formation of agarwood in Aquilaria sinensis trees in this study. In this study, pretreatment was carried out before exposing the Aquilaria sinensis trees to fungi. Moreover, isolated fungi were screened for resistance against benzyl acetone. The method presented here is able to narrow down the range of fungi that promote agarwood formation, and reduce the time and monetary cost of screening these fungi. The study would expand the library of fungi known for promoting agarwood formation.
30. Referencing for study not adequate.

Changes have been made in the text

Reviewer #2

1. Abstract need to be more summarized with the actual study results.

Changes have been made in the text
2. The introduction section needs to be adhered to with the proposed title. Also needs to be referred to relevant published literature. The following published articles might be helpful in the enhancements of the introduction section.
Changes have been made in the text
3. The novelty of the work needs to be highlighted. The objectives of the work can be elaborated for reproduction of the work.

Changes have been made in the text
4. Methods need to be reproducible. The authors provided too descriptive methods. I suggest shortening them and extra parts can be moved to the results section.

Changes have been made in the text
5. Also, the methods can be separated into sub-topics for ease of access. Like GCMS analysis and the different shock treatments.

Changes have been made in the text
6. For the GCMS analysis following published literature might be useful such as Gas chromatography analysis of the microwave-aided extracted agarwood oil from physically induced Aquilaria malaccensis trees in Northern Thailand. Maejo International Journal of Energy and Environmental Communication, 4(3), 52-55.

Changes have been made in the text
7. In the methods, the authors mentioned the PCR amplification, but in the results, I am unable to find the results related to it. No gel images or DNA analysis results were presented.

Changes have been made in the text.
8. If the authors plan to include Table 3 can be moved to supplementary information. AS Figure 5 shows the chromatographs.

Changes have been made in the text.
9. The results need to be enhanced with the help of literature. The discussion section is not comprehensive enough to refer to published articles for improvements.

Changes have been made in the text.
10. The grammatical errors and the English language need to be refined.

Changes have been made in the text.
