# Supplementary material for: Isolation and screening of fungi for enhanced agarwood formation in Aquilaria sinensis trees
Source: PLoS One. 2024 Jun 14;19(6):e0304946. doi: 10.1371/journal.pone.0304946 (PMC11178209; doi:10.1371/journal.pone.0304946)
Supplement: Attachment — Submitted filename: Comments for Manuscript_PLOS 15_12_2023.docx [file pone.0304946.s010.docx]

**Overall Comment:**

Though the Authors took the effort to modify the manuscript. Still there is a huge scope to modify it further to make it a much appreciable piece of work. A major revision is still needed to make it a scientifically presented manuscript.

**From the previous comments 3 comments where not answered adequately**

1. A thorough English check of the manuscript
2. Comments 15 Not adequately answered. A more insightful explanation needed for the amalgamation of too many methods (Physical, heat, electric current creating a state of over stress to the plant). Justification on the role of the pretreatment needed to be elaborated more scientifically in discussion
3. Comment 19: Not adequately answered.

**Abstract:**

1. Agarwood is formed when Aquilaria sinensis trees are harmed naturally or by humans: **Modify the statement.**
2. In this study, dominant fungal species were isolated and purified from the surfaces and electroshock holes of Aquilaria sinensis trees by fire and electroshock treatments: **Modify the statement**
3. In agarwood products formed by using Trichoderma sp. And Neurospora sp., the proportions of ether extracts were 50.22% and 48.71%, respectively, exceeding the 10% threshold specified by the Chinese Pharmacopoeia: **Modify to make it applicable to International Readers too. What is agarwood products? Its just raesin deposition.**
4. Fungal application shortened the time of natural agarwood formation: **If you have applied fungal inoculum how is its natural infection, its induced formation. Modify the statement.**

Introduction:

5. Fungi promoting the formation of agarwood are firstly tolerant to some extent to the active ingredients of agarwood: **Quote the reference for the statement.**
6. In this study, pretreatment was carried out before exposing the *Aquilaria sinensis* trees to fungi. Moreover, isolated fungi were screened for resistance…………………… The study would expand the library of fungi known for promoting agarwood formation: **These are concluding lines used by author in conclusion also. These cannot be part of Introduction segment.**
7. The method presented here is able to narrow down the range of fungi that promote agarwood formation, and reduce the time and monetary cost of screening these fungi. The study would expand the library of fungi known for promoting agarwood formation: **Two contradictory statements made in same para, Modify.**

**Materials and methods**

8. **Aquilaria sinensis needs to be italic**
9. The discolored wood of Aquilaria sinensis was collected after treatment by the electric shock pretreatment for 3 months
10. 1.5ml should be 1.5 ml. Follow international norms Value space SI Units.
11. -20℃ should be -20 ℃.
12. 5cm should be 5 cm. Follow this throughout the manuscript.
13. Fig.2 Map of the dominant colony on the surface of the tree and the dripping and sampling position of the bacterial liquid: **Should be Fig.2. Picture of the of the dominant colony on the surface of the tree, the dripping and sampling position of the fungal liquid.**
14. Evidences in form of Photographs are not adequate, some more detailing needed, e.g. pictures of inoculation with GI tag pictures etc.

**Results**

1. Using molecular identification, the dominant fungal strains on the surface of *Aquilaria sinensis* were initially identified as *Trichoderma sp.* and *Neurospora sp.*, which were labelled as LV and H, respectively: **Please provide the evidences of identification eg. Amplification of the PCR products (gel images), Phylogenetic analysis etc as supplementary data**
2. The red and green fungal colonies that covered the burnt parts of the trees after the fire treatment (Fig 2) were attributed to the decay and removal of other microorganisms from the trees due to fire, resulting in the vigorous growth of Trichoderma sp. and Neurospora sp.: **Is a very very vague statement. Either remove it or provide evidences for stating the same.**
3. **The sentences** “The red and green fungal colonies that covered the burnt parts of the trees after the fire treatment………………………………. intrinsic factors (such as chemical compositions) and extrinsic factors (such as the climate, light, rainfall and soils) may affect the parasitism and diversity of endophytic fungi in agarwood” **are not results need to be moved to discussion section.**
4. In the sentence “Promotion of agarwood formation in Aquilaria sinensis by artificial inoculation of benzyl acetone-tolerant fungi” ***Aquilaria sinensis* needs to be in italics.**
5. **The sentences** “The low sesquiterpene content in the agarwood……………………………………absence of chromone components in agarwood samples may be due to insufficient induction time” **are explanations to the results obtained and would be appropriate if are moved to discussion section.**
6. **Figure legends are poorly presented**

**Discussion:**

1. After injection, the fungal solution is mixed into the trunk sap, and then diffused into other parts of tree: **Is the statement correct**?

**Conclusion:**

1. **Needs further modification to highlight upon the novelty of the work. The continuity between the statements is missing, which the authors need to improve upon majorly.** **The conclusion needs to be rewritten.**
