# Supplementary material for: Isolation and screening of fungi for enhanced agarwood formation in Aquilaria sinensis trees
Source: PLoS One. 2024 Jun 14;19(6):e0304946. doi: 10.1371/journal.pone.0304946 (PMC11178209; doi:10.1371/journal.pone.0304946)
Supplement: Attachment — Submitted filename: Response to Reviewers.docx [file pone.0304946.s012.docx]

Response to Reviewers

According to the review comments from editors, this paper has an overall problem with the English language, so I made a complete revision of my manuscript to meet the journal requirements.

From the previous comments 3 comments where not answered adequately

1. **A thorough English check of the manuscript**

The manuscript has been thoroughly reviewed in English and revised.

2. **Comments 15 Not adequately answered. A more insightful explanation needed for the amalgamation of too many methods (Physical, heat, electric current creating a state of over stress to the plant). Justification on the role of the pretreatment needed to be elaborated more scientifically in discussion**

I have added the understanding of the pretreatment effect in the discussion.

3. **Comment 19: Not adequately answered.**

It has been changed in the new manuscript, previously meaning that after ether extraction, the material finally extracted the yield of the essential oil.

Abstract:

1. **Agarwood is formed when Aquilaria sinensis trees are harmed naturally or by humans: Modify the statement.**

The statement has been revised

2. **In this study, dominant fungal species were isolated and purified from the surfaces and electroshock holes of Aquilaria sinensis trees by fire and electroshock treatments: Modify the statement**

The statement has been revised

3. **In agarwood products formed by using Trichoderma sp. And Neurospora sp., the proportions of ether extracts were 50.22% and 48.71%, respectively, exceeding the 10% threshold specified by the Chinese Pharmacopoeia: Modify to make it applicable to International Readers too. What is agarwood products? Its just raesin deposition.**

The statement has been revised.In this paper, the agarwood product means that the discolored wood obtained on the tree through the action of fungi is identified as containing agarwood ingredients after the chemical composition. The wood is the agarwood product.

4. **Fungal application shortened the time of natural agarwood formation: If you have applied fungal inoculum how is its natural infection, its induced formation. Modify the statement.**

The statement has been revised

Introduction:

5. **Fungi promoting the formation of agarwood are firstly tolerant to some extent to the active ingredients of agarwood: Quote the reference for the statement.**

**Have already cited reference**

6. **In this study, pretreatment was carried out before exposing the *Aquilaria sinensis* trees to fungi. Moreover, isolated fungi were screened for resistance…………………… The study would expand the library of fungi known for promoting agarwood formation: These are concluding lines used by author in conclusion also. These cannot be part of Introduction segment.**

Have deleted

7. **The method presented here is able to narrow down the range of fungi that promote agarwood formation, and reduce the time and monetary cost of screening these fungi. The study would expand the library of fungi known for promoting agarwood formation: Two contradictory statements made in same para, Modify.**

Has been modified

**Materials and methods**

8. **Aquilaria sinensis needs to be italic**

Has been modified

9. **The discolored wood of Aquilaria sinensis was collected after treatment by the electric shock pretreatment for 3 months**

Has been modified

10. **1.5ml should be 1.5 ml. Follow international norms Value space SI Units.**

Has been modified

11. **-20℃ should be -20 ℃.**

Has been modified

12. **5cm should be 5 cm. Follow this throughout the manuscript.**

Has been modified

13. **Fig.2 Map of the dominant colony on the surface of the tree and the dripping and sampling position of the bacterial liquid: Should be Fig.2. Picture of the of the dominant colony on the surface of the tree, the dripping and sampling position of the fungal liquid.**

Has been modified

14. **Evidences in form of Photographs are not adequate, some more detailing needed, e.g. pictures of inoculation with GI tag pictures etc.**

More detailed information has been added to the attachment

**Results**

1. **Using molecular identification, the dominant fungal strains on the surface of *Aquilaria sinensis* were initially identified as *Trichoderma sp.* and *Neurospora sp.*, which were labelled as LV and H, respectively: Please provide the evidences of identification eg. Amplification of the PCR products (gel images), Phylogenetic analysis etc as supplementary data**

The alignment information of the sequencing results has been provided in the attachment

2. **The red and green fungal colonies that covered the burnt parts of the trees after the fire treatment (Fig 2) were attributed to the decay and removal of other microorganisms from the trees due to fire, resulting in the vigorous growth of Trichoderma sp. and Neurospora sp.: Is a very very vague statement. Either remove it or provide evidences for stating the same.**

Has been modified

3. **The sentences “The red and green fungal colonies that covered the burnt parts of the trees after the fire treatment………………………………. intrinsic factors (such as chemical compositions) and extrinsic factors (such as the climate, light, rainfall and soils) may affect the parasitism and diversity of endophytic fungi in agarwood” are not results need to be moved to discussion section.**

Has been modified

4. **In the sentence “Promotion of agarwood formation in Aquilaria sinensis by artificial inoculation of benzyl acetone-tolerant fungi” *Aquilaria sinensis* needs to be in italics.**

Has been modified

5. **The sentences “The low sesquiterpene content in the agarwood……………………………………absence of chromone components in agarwood samples may be due to insufficient induction time” are explanations to the results obtained and would be appropriate if are moved to discussion section.**

Has been modified

6. **Figure legends are poorly presented**

Has been modified,But I don't know how to modify it even better

Discussion:

1. **After injection, the fungal solution is mixed into the trunk sap, and then diffused into other parts of tree: Is the statement correct?**

I think correctly, I inject the fluid into the trunk and definitely spread to the rest of the tree. Maybe the process is not very accurate, but the result should be what I said.

Conclusion:

1. **Needs further modification to highlight upon the novelty of the work. The continuity between the statements is missing, which the authors need to improve upon majorly. The conclusion needs to be rewritten.**

Has been modified

**Finally, I wish you a merry Christmas, if there are any deficiencies, please understand, I wish you a happy life, everything goes well.**
