# Supplementary material for: Isolation and screening of fungi for enhanced agarwood formation in Aquilaria sinensis trees
Source: PLoS One. 2024 Jun 14;19(6):e0304946. doi: 10.1371/journal.pone.0304946 (PMC11178209; doi:10.1371/journal.pone.0304946)
Supplement: Attachment — Submitted filename: Response to Reviewers.docx [file pone.0304946.s014.docx]

**Response to Reviewers**

**Abstract:**

1. **Agarwood is not produced naturally; it only forms after natural or man-made damage to Aquilaria sinensis. Replace man-made damage with suitable wording**

Modifications have been made

**Introduction:**

1. **The naturally occurring agarwood has long outstripped the market demand, and at the end of the..Replace outstripped with outrun**

Modifications have been made

2. **Benzyl acetone is a representative compound of the aromatic content in agarwood……….the statement needs to be in context. Here it is just a hanging statement.**

Modifications have been made

**Results**

1. **Screening of benzyl acetone-tolerant fungi : a certain extent, the isolated and purified fungal strains were screened for tolerance to benzyl acetone.**

Modifications have been made

2. **GC-MS detection of agarwood samples ……where is th evidence for the same provided.**

Original documentation of the GC-MS test has been provided.

3. **The agarwood products…..use statement “The agarwood”**

Modifications have been made

4. **Fig.3 Agarwood samples…………Reasin deposition seen in figure is very very less than claimed in the text. Please explain.**

The image has been replaced.As can be seen in the supplementary document (Schematic representation of the fungal inoculation with Aquilaria sinensis)

5. **For the query Using molecular identification, the dominant fungal strains on the surface of Aquilaria sinensis were initially identified as Trichoderma sp. and Neurospora sp., which were labelled as LV and H, respectively: Please provide the evidences of identification eg. Amplification of the PCR products (gel images), Phylogenetic analysis etc as supplementary data**

Gel images and phylogenetic trees of PCR amplification products of H and LV have been provided in the Supplementary Material

6. **The alignment information of the sequencing results has been provided which I think is not adequate, please provide the gel images etc.**

Gel images and phylogenetic trees of PCR amplification products of H and LV have been provided in the Supplementary Material
