# Supplementary material for: Isolation and screening of fungi for enhanced agarwood formation in Aquilaria sinensis trees
Source: PLoS One. 2024 Jun 14;19(6):e0304946. doi: 10.1371/journal.pone.0304946 (PMC11178209; doi:10.1371/journal.pone.0304946)
Supplement: Attachment — Submitted filename: Response to Reviewers2.docx [file pone.0304946.s015.docx]

**Response to Reviewers**

1. **Figure 2 and Figure 3 descriptions can be more specific. For figure 2, specifically mention which inoculation method results in agarwood formation shown in the photograph. For figure.3 specifically mention the type of sample used for GC MS analysis for each. Not in the form of Note. If abbreviations are used then also provide full form in figure captions.**

The left side of Fig. 2 shows the dominant fungi in the tree after fire treatment. After isolating and purifying the fungi, the fungal solution was injected into the tree using the method shown in the right side of the figure, so it is the fungal injection method shown in the right side of the figure that induces the formation of agarwood. This is explained in "Artificial inoculation of fungi to induce the formation of agarwood in Aquilaria sinensis".

For Figure 3, the type of each sample used for GC MS analysis has been described. **2. Regarding methodology used for GC-MS analysis reference 20 is cited, for which it is mentioned that full text is only available in Chinese. Give complete details of GC-MS methods used in the study in the manuscript**

Already revised in the manuscript
